# Anti-Epileptic Effect of Ganoderma Lucidum Polysaccharides by Inhibition of Intracellular Calcium Accumulation and Stimulation of Expression of CaMKII α in Epileptic Hippocampal Neurons

**DOI:** 10.1371/journal.pone.0102161

**Published:** 2014-07-10

**Authors:** Shu-Qiu Wang, Xiao-Jie Li, Hong-Bin Qiu, Zhi-Mei Jiang, Maria Simon, Xiao-Ru Ma, Lei Liu, Jun-Xing Liu, Fang-Fang Wang, Yan-Feng Liang, Jia-Mei Wu, Wei-Hua Di, Shaobo Zhou

**Affiliations:** 1 Department of Pathophysiology, School of Basic Medical Sciences, Jiamusi University, Jiamusi, P. R. China; 2 Children Neural Rehabilitation Laboratory of Jiamusi University, School of Rehabilitation Medical Sciences, Jiamusi University, Jiamusi, Heilongjiang Province, P. R. China; 3 School of Public Health, Jiamusi University, Jiamusi, Heilongiang Province, P.R. China; 4 Department of Life Science, Institute of Biomedical and Environmental Science and Technology, University of Bedfordshire, Luton, United Kingdom; Oregon Health & Science University, United States of America

## Abstract

**Purpose:**

To investigate the mechanism of the anti-epileptic effect of Ganoderma lucidum polysaccharides (GLP), the changes of intracellular calcium and CaMK II α expression in a model of epileptic neurons were investigated.

**Method:**

Primary hippocampal neurons were divided into: 1) Control group, neurons were cultured with Neurobasal medium, for 3 hours; 2) Model group I: neurons were incubated with Mg^2+^ free medium for 3 hours; 3) Model group II: neurons were incubated with Mg^2+^ free medium for 3 hours then cultured with the normal medium for a further 3 hours; 4) GLP group I: neurons were incubated with Mg^2+^ free medium containing GLP (0.375 mg/ml) for 3 hours; 5) GLP group II: neurons were incubated with Mg^2+^ free medium for 3 hours then cultured with a normal culture medium containing GLP for a further 3 hours. The CaMK II α protein expression was assessed by *Western*-blot. Ca^2+^ turnover in neurons was assessed using Fluo-3/AM which was added into the replacement medium and Ca^2+^ turnover was observed under a laser scanning confocal microscope.

**Results:**

The CaMK II α expression in the model groups was less than in the control groups, however, in the GLP groups, it was higher than that observed in the model group. Ca^2^^+^ fluorescence intensity in GLP group I was significantly lower than that in model group I after 30 seconds, while in GLP group II, it was reduced significantly compared to model group II after 5 minutes.

**Conclusion:**

GLP may inhibit calcium overload and promote CaMK II α expression to protect epileptic neurons.

## Introduction

There are around 60 million epileptic patients worldwide [1], and nearly 80% of them are in developing regions [2], [3]. It is a condition that can seriously affect quality of life and a global campaign against epilepsy is required [4]. Epilepsy can be managed by drugs, for example, 75% of patients respond well to phenobarbital treatment [5]. After one year of treatment with sodium valproate, 42% patients were seizure free and 84% patients had a decrease in seizure frequency of, at least, 50% [6]. However, most of drugs have side-effects on brain function, e.g. mood alteration or neurocognitive function [2], [5], reduction in neuron excitation and inhibition of normal activity. Thus, finding a more effective drug with fewer side effects is of continuing importance [7].

Epilepsy is characterized by the occurrence of spontaneous, recurrent, unprovoked seizure discharges and high-frequency firing of neuronal populations in the central nervous system [8]. Calcium channels play a critical role in neuronal firing and circuit excitability [8], [9]. The accumulation of elevated levels of calcium, a major signaling molecule in the central nervous system, in neurons is regarded as one of the characteristics of epilepsy. This has been shown in both *in vivo* [8], [9] and *in vitro* studies [10], [11]. Therefore, maintaining the homeostasis of calcium ions is an important target in the prevention and treatment of epilepsy [11]–[13]. It has been demonstrated that when free intracellular calcium concentration (Ca^2+^i) is reduced, via lower extracellular Ca^2+^ environments or using Ca^2+^i chelating agents, development of epilepsy is prevented [14]–[16].

Ca2+/calmodulin-dependent protein kinase II (CaMK II), is a protein kinase involved in the regulation of neuronal activity in various ways, including neurotransmitter synthesis and release, and activity-dependent neuronal modifications [17]–[19]. CaMK II α factor plays an important role in Ca^2^^+^ transfer in many types of neurons. It can bind Ca^2+^ and forms Ca^2+^/CaM complexes [20]–[22]. CaMK II inactivation is associated with many experimental models of epilepsy [23], [24].

Ganoderma lucidum has been used as an effective drug for centuries in East Asia. It has been a popular traditional drug to treat various human diseases, such as hepatitis, hypertension, hypercholesterolemia and cancer [25]–[27]. Recent efforts from our team have shown that Ganoderma lucidum, its spore (GLS) [28]–[33], has anti-epileptic effects in *in vivo* and *in vitro* studies. GLS has been shown to increase aminobutyric acid and decrease amino glutaminic acid [29]; stimulate Cav-1, neurotrophin-4 expression and inhibit NF-kB and N-Cadherin expression [31], [33]. However, there is no research regarding the effect of GLP on Ca^2+^ turnover or on CaMK II α expression in epileptic neurons.

To further investigate the mechanism of GLP in the treatment of epilepsy, calcium turnover and CaMK II α expression were analysed in a model of epileptiform discharge of hippocampal neurons treated with GLP. This model has been developed and widely used to test the efficacy and mechanism of anti-epilepsy drugs [11], [33], [34]. In this study, 1) primary hippocampal neurons isolated from <1 day old rats were cultured up to 14 days to identify their purity, the highest purity of hippocampal neurons at day 9 were harvested and used for this study; 2) these were then cultured in a medium with or without GLP. The Ca^2^^+^ turnover and CaMK II α expression in epileptiform neurons were investigated.

## Materials and Methods

### 2.1. Animals

Newborn Wistar rats (less than 24 hours old) were purchased from the Animal Center of Jiamusi University. The animal study was approved by the animal Ethical Committee of JiaMusi University and carried out in strict accordance with the guidelines of the animal ethical committee of JiaMusi University. All surgery was performed under sodium pentobarbital anesthesia, and all efforts were made to minimize suffering.

### 2.2. Culture and identification of primary hippocampal neurons

Hippocampal tissues from rats were harvested using conventional methods [33]. Briefly, rat brain was removed under sterile conditions into D-Hanks solution at 4°C. Hippocampal tissues were collected under microscope and cut into 1 mm^3^ pieces which were then incubated with 0.125% trypsin (total volume was about 5 times volume of hippocampal tissues) in a 37°C incubator (containing 5% CO_2_),with gentle shaking of the preparation every 5 minutes. 20 minutes later,an equivalent volume of maintaining medium [Neurobasal medium (Cat. No. 21103049, Gibco), 2% B27 supplement (Cat. No. 17504044, Gibco) and 0.5 mmol/L glutamine] was added and the preparation incubated for a further 5 minutes to stop the trypsin digestion. The cells were then centrifuged at 1000 rpm for 5 minutes. Supernatant was removed and maintaining medium added to the cells which were then filtered through a 200 µM mesh. The filtered solution with cells was adjusted to a 5×10^5^/ml cell suspension by using maintaining medium. 6 ml, 0.2 ml, 1 ml and 2 ml of the above cell suspension was transferred into 100 ml culture flasks, each well of a 96-well plate, a 24-well plate or a 6-well plate respectively. Cultures were incubated in a 37°C incubator (containing 5% CO_2_) for 24 hours, after which time, the whole culture medium [Neurobasal medium, 2% B27 supplement, 0.5 mmol/L glutamine and 10% FBS] was replaced with the nutrient maintaining medium. Half the volume of medium was changed every other day. 24 well and 6 well plates containing cover slides were treated with 0.1 mg/ml poly-L-lysine.

Hippocampal neurons were identified by detection of neuron specific enolase (NSE), a protein specifically expressed in hippocampal neurons. This was done using day 9 cells incubated in a 6 well plate with anti-NSE antibody (Cat. No BA0535, Wuhan Boster Bio-Engineering Ltd, China) and secondary antibody labeled with FITC (Cat. No. BA1105, Wuhan Boster Bio-Engineering Ltd, China). Detailed methods have been reported previously [33].

### 2.3 Evaluation of the purity of hippocampal neurons

Hippocampal neurons were cultured to a density of 2.5×10^4^/ml. Five culture flasks were used respectively for day 5, 7, 9, 11 and 14. The number of hippocampal neurons and glial cells was counted in selected 10 visual fields under a 100× microscope. The purity of hippocampal neurons was calculated by using the following formula: the number of hippocampal neurons/(the number of hippocampal neurons + the number of glial cells).

### 2.4 Establishment of the epileptiform discharge hippocampal neuron model

The epileptic cell model was set up using a conventional method [11], [33], [34]. Hippocampal neurons were cultured in nutrient maintaining medium. At day 9, the nutrient maintaining medium was replaced with extracellular medium without Mg^2+^ (145 mmol NaCl, 2.5 mmol KCl, 2 mmol CaCl_2_, 10 mmol HEPES, 10 mmol glucose, 0.002 mmol glycine, pH 7.2, 290±10 mOsm) and treated for 3 hours. This treatment induces permanently manifested recurrent, spontaneous seizure discharges characteristic of the same electrographic properties seen in human epilepsy [35]. Then, the normal extracellular culture medium (145 mmol NaCl, 2.5 mmol KCl, 2 mmol CaCl_2_, 1 mmol MgCl_2,_ 10 mmol HEPES, 10 mmol glucose, 0.002 mmol glycine, pH 7.2, 290±10 mOsm) was replaced and the cells incubated for a further 3 hours.

### 2.5 GLP sterilisation processing

GLP was purchased from Baoding Shida Biotechnological Inc, Hebei Province, China (Cat. RM081215). GLP sterilization was undertaken by microporous membrane filtration. 0.1 g of GLP was weighed and put into 10 ml nutrient maintaining medium, in a centrifuge tube, was dissolved by thorough mixing and filtered through a 0.22 µm filter into another sterile centrifuge tube. The tube was sealed and stored at 4°C. Aliquots of the solution were added into flasks containing culture medium and incubated for 6 h, 12 h, 24 h, 48 h and 72 h at 37 °C. No bacterial or fungal growth was observed under microscope.

### 2.6 Determination of maximum non-toxic concentration of GLP and appropriate concentration

A 10 mg/ml of GLP suspension was added into maintaining nutrient medium and the solutions were diluted into 12 different concentrations (1:5, 1:10, 1:20, ……). 0.1 ml of each concentration was put into 8 wells of a 96-well plate containing, n = 4, 0.2 ml hippocampal neurons (5×10^4^/ml cell suspension) and cultured for 7 days. A control group with normal cells was also set up. The 96-well plates were incubated in an incubator at 37°C with 5% CO_2_ for 24 hours. Then, 20 µl of 5 mg/ml MTT (freshly made) was added into each well and the plates were incubated for a further 4 hours. The medium was removed and 200 µl of DMSO was added into each well and the plates were put onto a gentle shaker for 10 minutes. The OD value was measured at 490 nm. The blank value was measured by using the control cells, with the same concentration of chemicals and culture medium, MTT and DMSO. The sample zero reading value was obtained by using culture medium, MTT and DMSO. The maximum nontoxicity concentration, 0.375 mg/ml, was found and all subsequent experiments were performed at this concentration based on this measurement.

### 2.7 Effect of GLP on intracellular Ca^2^^+^ accumulation and CaMK II α expression

#### 2.7.1 Effect of GLP on Ca^2+^ turnover during the establishment of the model

The neurons cultured at day 9 were used for the following experiments, n = 4, (1) Control group I: nutrient maintaining medium was replaced with extracellular medium; (2) Model group I: nutrient maintaining medium was replaced with Mg^2+^ free extracellular medium; (3) GLP group I: nutrient maintaining medium was replaced with Mg^2+^ free medium containing GLP (0.375 mg/ml). Fluo-3/AM (FluoroPure grade, Life technology, Cat, F-23915) was added into the replacement medium discussed above immediately after replacing the nutrient maintaining medium [36]. Ca^2+^ turnover was observed/recorded for 10 minutes continuously under a laser scanning confocal microscope.

#### 2.7.2 Effect of GLP on Ca^2+^ turnover during treatment

Neurons cultured at day 9 were used for the following experiments, n = 4, (1) Control group II: nutrient maintaining medium was replaced with extracellular medium for 3 hours. Then, extracellular medium was replaced with nutrient maintaining medium containing Fluo-3/AM [36]; (2) Model group II: nutrient maintaining medium was replaced with Mg^2+^ free extracellular medium for 3 hours. Then, the Mg^2+^ free extracellular medium was replaced with nutrient maintaining medium with Fluo-3/AM; (3) GLP group II: nutrient maintaining medium was replaced with Mg^2+^ free medium for 3 hours. This medium was then replaced with nutrient maintaining medium containing GLP (0.375 mg/ml) and Fluo-3/AM. Ca^2+^ turnover was observed/recorded in 30 neurons, immediately following addition of Fluo-3/AM to the medium, under a laser scanning confocal microscope.

#### 2.7.3 Expression of CaMK II α in neurons by confocal laser microscope and Western-blot

Neurons cultured to day 9 were used for the following experiment, n = 4. Neurons were divided into (1) Control group III: nutrient maintaining medium was replaced with extracellular medium for 3 hours, then extracellular medium was replaced with normal maintaining medium and cells incubated for a further 2 hours; (2) Model group III: normal maintaining medium was replaced with Mg^2+^ free extracellular medium for 3 hours, then replaced with normal maintaining medium and cells incubated for 2 hours; (3) GLP group III: normal maintaining medium was replaced with Mg^2+^ free medium containing GLP (0.375 mg/ml) for 3 hours, then replaced with normal maintaining medium and cells incubated for 2 hours; (4) GLP group IV: normal maintaining medium was replaced with Mg^2+^ free medium for 3 hours, then cells cultured with a normal maintaining medium containing GLP for a further 2 hours.

Neurons from the above experimental conditions were washed for 5 minutes, 3 times with0.01 mol PBS. The neurons were then fixed with 4% paraformaldehyde for 30 minutes and then washed 3 times (5 minutes per wash) with 0.01 mol PBS. Neurons were then incubated with 0.5% Triton-100 for 20 minutes, followed by washing 3 times, 5 minutes per wash, with 0.01 mol PBS. Next, the neurons were incubated with 10% goat serum blocking solution (WuhanBoster Lt.d, China) at room temperature for 30 minutes. Following removal of the blocking solution, rabbit anti-mouse CAMK II α antibody (WuhanBoster Ltd, China), at a final dilution 1:100, was added and incubated at 4°C, overnight. The neurons were then moved to room temperature for 2 hours, followed by washing 3 times, 5 minutes per wash, with 0.01 mol PBS. Under dark conditions, goat anti-rabbit FITC fluorescence secondary antibody (final dilution 1:50) was added and incubated at room temperature for 2 hours. This was then rinsed with 0.01 mol PBS three times, 5 minutes per wash. A drop of saline was applied to a slide, the coverslip with the neurons was removed from the 6-well plate, and placed with the neurons “facedown” into the saline on the slide. In each group, n = 4, 30 neurons were randomly observed under a laser scanning confocal microscope and images were taken. The fluorescence intensity was calculated by using Image-Pro Plus 6.0 software.

Proteins were prepared using commercial lysis buffer (Cat. No. P0013, Beyotine Institute Biotechnology, China) according to the product protocols. Protein sample was mixed with 5× loading buffer solution, denatured for 5 minutes at 100°C, and then subjected to 10% sodium dodecylsulfate-polyacrylamide gel electrophoretic separation followed by transferring the protein band onto a nitrocellulose filter. The nitrocellulose filter was blocked with 1% bovine serum albumin overnight. It was then incubated with a rabbit-anti-β-actin polyclonal antibody (BAB) and rabbit-anti-CaMK II α (Cat. No. BA2305, PA-1497, Wuhan Boster Bio-Engineering Ltd, China) respectively for 2 hours at 37°C, rinsed with tris-buffered saline with Tween 20 (TBST) 3 times for 10 minutes each, and then incubated with horse radish peroxidase-labeled goat-anti-rabbit IgG antibody for 2 hour at 37°C. The blot was then rinsed twice for 10 minutes with TBST solution, rinsed one more time with TBS solution for 10 minutes, and finally exposed in a X film and the density of the protein band was analysed.

### 2.8. Statistical Analysis

Data were shown by mean±standard deviation and the results were analyzed using Microsoft Excel 2007 and SPSS18.0. Tukey’s test following one-way ANOVA was used. There was significant statistical difference when *P*<0.05.

## Results

### 3.1. Characterization of the hippocampal neuron

The morphology of hippocampal neurons was recorded at 24 hours (Image S1A), day 3 (Image S1B), day 5 (Image S1C), and day 9 (Image S1D). At day 9, the neurons became mature and aggregated into clumps, the whole growth of the neurons was unevenly distributed, and a single neuron was difficult to identify. The neurons were identified by NSE which showed that the somas of the hippocampal neurons were plump, triangular, round, fusiform or polygonal. The neurites were thick and interweaved into a network. The cytoplasm and the neurites were green revealing the presence of NSE, while the nuclei were stainless [33]. The hippocampal neurons cultured under low-density conditions were observed under the microscope. The hippocampal neurons at day 5, 7, 9, 11 and 14 was 33.13±11.18, 63.03±17.73, 83.16±23.85, 67.09±18.81, 80.94±17.78 (n = 5 for each sample; neuron cells were counted in 10 visual fields under a 100× microscope), with purity of 88%; 91%; 96%; 96%; and 96% respectively. At day 11 and 14, the hippocampal neurons grew well compared with day 9, however, the purity of the neurons was not obviously changed. Thus, hippocampal neurons cultured at day 9 were used in this study.

### 3.2 Effect of GLP on the concentration of Ca^2^^+^ in epileptic hippocampal neurons

**Figure 1:**
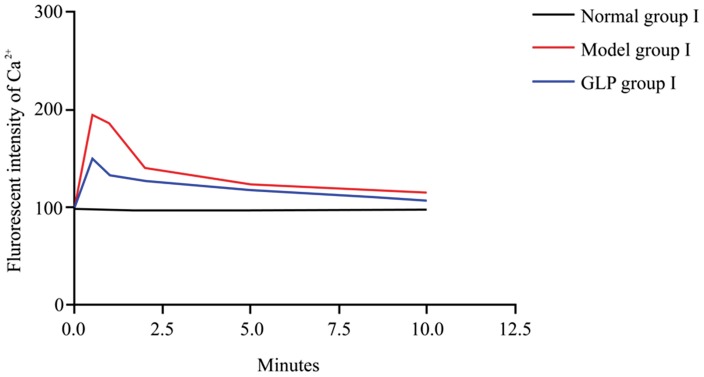
Changes in fluorescence intensity of Ca^2+^ in neurons during establishment of the model, n = 4, thirty neurons were observed in each replicate. There is a significant increase (within 30 seconds) in fluorescence intensity of Ca^2+^ in neurons in the model group compared to the control group (p<0.001). This increase also occurs in the GLP I group, and it decreases in both Model and GLP I groups with time up to 10 minutes when the Model group is still significantly higher than the Control group I, but, there is no difference between the Control group I and GLP I groups at 10 minutes. Tukey’s test following one way ANOVA was used to perform statistical analysis.

Results showed that Ca^2^^+^ was located in the cytoplasm and nucleus (Image S2). Ca^2^^+^ fluorescence intensity (196±9, Table 1) in the hippocampal neurons of model I (Image S2B) was significantly higher than that observed in the Control group I (98±11, P<0.001, Table 1 and Image S2A). Mg^2+^ free extracellular fluid significantly increased Ca^2^^+^ in the cytoplasm in the neurons with Ca^2^^+^ levels reaching a peak around 30 s (Figure 1). However, when GLP was added into the Mg^2+^ free extracellular fluid, Ca^2^^+^ fluorescence intensity in the hippocampal neurons decreased to 151±9 (P<0.001) (Image S2C). This indicates GLP inhibits Ca^2^^+^ accumulation in the cytoplasm of neurons caused by Mg^2+^ free medium. Calcium levels in the cytoplasm and nucleus of neurons from GLP group I and Model group I dropped soon after 30 s (Figure 1, Table 1). Although the fluorescence intensity in both Model I and GLP I groups was higher than the Control group I, the GLP group I was always lower than that of Model group I (Table 1 and Figure 1).

**Table 1:** Changes in fluorescence intensity of Ca^2+^ in neurons during establishment of the model.

Groups	0 min	0.5 min	1 min	2 min	5 min	10 min
Control group I	99±9	98±11	99±10	97±10	97±8.7	98±10
Model group I	99±8	196±9^##^	186±11^##^	141±8^##^	123±7^##^	115±8^#^
GLP group I	99±10	151±9^##^**	133±11^##^**	127±9^##^	118±6^##^	108±8

Note: Data is mean±SD, total fluorescence intensity were recorded in 30 neurons from 4 samples of each group; Tukey’s test following ANOVA was used; Compared to Control group I, ^##^ *P*<0.001; ^#^ *P*<0.01; Compared to Model group I, **P<0.001.

**Figure 2:**
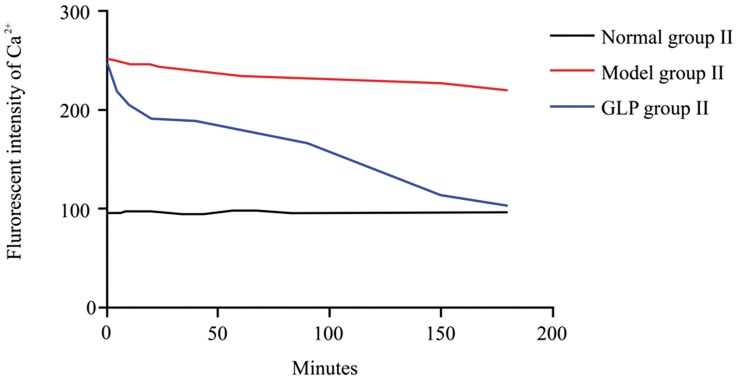
Changes in fluorescence intensity of Ca^2+^ in neurons during the treatment, n = 4, thirty neurons were observed in each replicates. There is no change in fluorescence intensity of Ca^2+^ in neurons in the Control group II during a 3 hour period. Fluorescence intensity of Ca^2+^ in neurons was highest in neurons cultured in Mg^2+^ ion free medium for 3 hours prior to replacement with normal maintaining medium i.e. Model group II; there is no significant decrease (P>0.05) between the zero time point (0 hour) and 3 hours following the normal medium replacement. However, following GLP treatment, there is a gradual decrease in Fluorescence intensity of Ca^2+^ in neurons, with levels almost returning to normal after 3 hours. Tukey’s test following one way ANOVA was used to perform statistical analysis.

Calcium intensity (253±21) in the neurons of Model group II was significantly higher compared to that seen in the Control group II (97±9) (*P*<0.01). There was no significant decrease even 3 hours after the replacement of normal maintaining medium (Image S3A and S3B). However, calcium intensity was significantly decreased only 5 minutes after replacement with a medium containing GLP in GLP group II (from 249±19 to 219±16, *P*<0.01). Calcium intensity, at 180 minutes, in the neurons of GLP group II (104±16, Image S3D) was still significantly lower than that observed in the Model group II (221±19) (Image S3B), but it was still significantly higher than that observed in the Control group II (97±11) after 3 hours treatment (Table 2, Figure 2).

**Table 2:** The fluorescence intensity of Ca^2+^ in neurons during GLP treatment.

Groups	0 min	5 min	10 min	20 min	40 min	60 min	90 min	120 min	150 min	180 min
Control group II	97±9	96±9	98±9	98±10	95±8	100±10	96±9	95±9	97±11	97±11
Model group II	253±21^##^	251±15^##^	247±9^##^	246±12^##^	241±16^##^	236±17^##^	233±11^##^	230±16^##^	227±21^##^	221±19^##^
GLP group II	249±19^##^	219±16^##^*	205±10^##^**	193±11^##^**	189±14^##^**	180±19^##^**	167±11^##^**	118±6^##^**	114±18**	104±16**

Note: Data is mean±SD, total fluorescence intensity were recorded in 30 neurons from 4 samples of each group; Tukey’s test following ANOVA was used; Compared to Control group II, ^##^ *P*<0.01; Compared to Model group II, **P<0.01; *P<0.05.

### 3.3 Effect of GLP on CaMK II α expression

**Figure 3:**
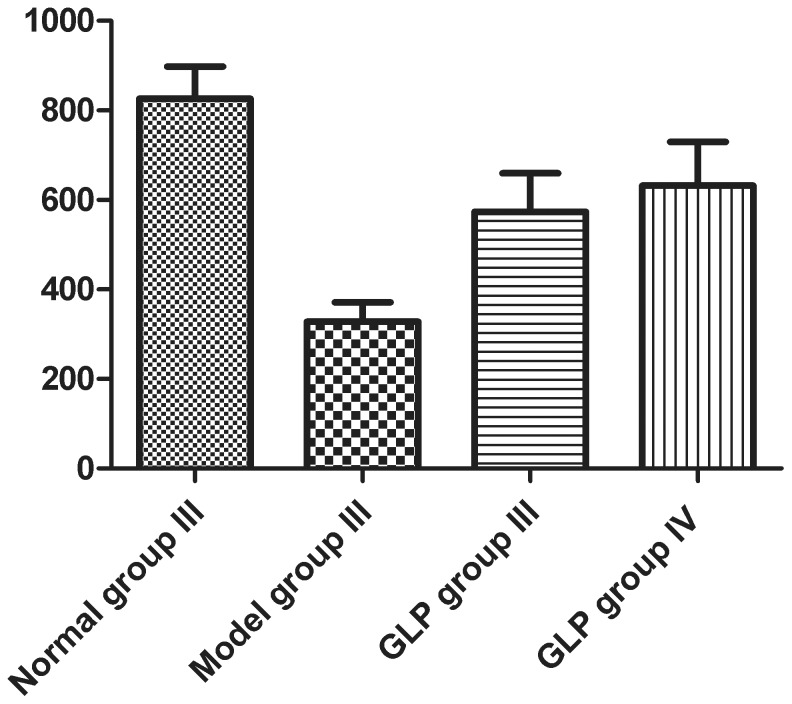
The fluorescence intensity of CaMK II α protein in hippocampal neurons in Control group III (A), Model group III (B), GLP group III (C) and IV (D). Data shown as mean±SD, n = 4. Corresponding, representative images are shown in Images S4A, S4B, S4C and S4D. Tukey’s test following one way ANOVA was used to perform the statistical analysis. #compared to Control group III, p<0.01; *compared to Model group III, p<0.01.

CaMK II α protein was expressed in normal and epileptic hippocampal neurons (Figure 3, Image S4, A–D). However, the fluorescence intensity (328±44, n = 4) of CaMK II α in Model group III (Image S4B) was significantly lower compared to that seen in the Control group III (826±70, n = 4, Image S4A) (*P*<0.01);GLP treatment can increase the CaMK II α expression in epileptic hippocampal neurons which was observed in both GLP group III (574±85, n = 4, Image S4C) and GLP group IV (632±98, n = 4, Image S4D) treated cells, but, the levels seen were still both lower than that observed in Control group III (Figure 3).

**Figure 4:**
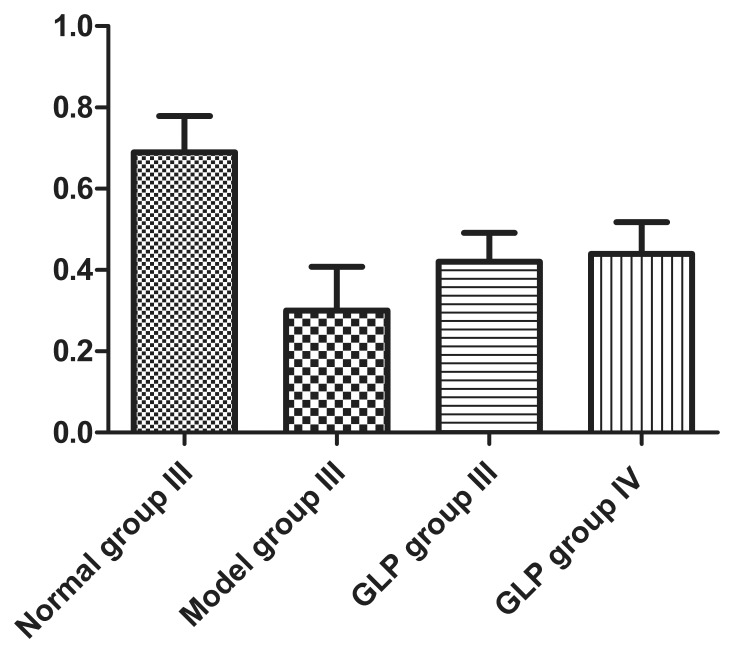
CaMK II α protein expression. Relative amount of CaMK II αprotein expressed in neurons in different groups (A) and *Western* blot analysis (B) of neurons from each untreated/treated group. Data in figure 4A is shown as mean±SD, n = 4. Tukey’s test following one way ANOVA was used to perform the statistical analysis. #indicated p<0.01, compared to Control group III; *indicated p<0.01, compared to Model group III.

CaMK II α protein was also detected in each group by *Western* blot (Figure 4B). CaMK II α protein expression in Model group III (0.30±0.11, n = 4) was lower than that seen in Control group III treated cells (0.69±0.09, n = 4, *P*<0.01); CaMK II α protein levels in GLP group III (0.42±0.07, n = 4) and IV (0.44±0.08, n = 4) were both lower than expression levels in Control group III (both P<0.01) but higher than that seen in Model group III (both P<0.05) (Figure 4A).

## Discussion

Ganoderma lucidum [32], [33], has been shown to be effective in the treatment of epilepsy in our previous studies. One of the mechanisms behind epilepsy is abnormal calcium homeostasis in neurons [11]. CaMK II α factor plays an important role in Ca^2^^+^ information transfer in many types of neurons. It can bind Ca^2+^ and form Ca^2+^/CaM complexes [22]. Many experimental models of epilepsy reveal CaMK II inactivation occurs during seizure activity and precedes neuronal cell death [24], [37]–[40]. In order to elucidate the mechanism of GLP prevention or treatment of epilepsy, intracellular calcium and expression of CaMK II α expression were measured in GLP treated models of epileptiform discharge of hippocampal neurons. This study was the first to show GLP might inhibit calcium overload and promote CaMK IIα expression to protect neurons cultured with Mg^2+^ free medium for 3 hours.

### 4.1. A model of epileptic hippocampal neurons

Excessive synchronized neuronal discharge, caused by abnormal calcium accumulation, is a primary characteristic of epilepsy. Although epileptic animal models have been used to test antiepileptic drugs and explore their mechanism of action, an *in vitro* model of epileptic hippocampal neurons has been widely accepted [34], [41]. In this model of epileptic hippocampal neurons, the culture medium with or without serum was used. Serum medium provides sufficient nutrients for cell growth, however, the diverse milieu of components in the serum affects the experimental results and interpretation. Culture medium without serum permits defined analysis of the experimental conditions. A previous study [42] compared the effects of different culture mediums (DMEM, DMEM/F12 and Neurobasal medium) on hippocampal neurons with the results showing increased longevity of neurons that were cultured in Neurobasal medium compared to those cultured with DMEM, or DMEM/F12. Furthermore, hippocampal neurons cultured with Neurobasal medium grow well at day 12, have stable cell membranes and mature synapses [43]. On the basis of this, the current study was carried out using hippocampal neurons cultured with Neurobasal medium. The morphology of neurons was observed up to 14 days using the method described previously [33]. The results showed that day 9 hippocampal neurons possess a mature morphology with a thick cell body, long axons, and a purity rate of 96±2.5 (%), meeting the experimental requirements.

In this model of hippocampal neurons cultured in a medium without Mg^2+^, the NMDA receptor [21], GABAA receptors [44] and voltage-dependent Ca^2+^ channel [45] will be activated, and these will increase the exterior calcium concentration around the neurons and thus increase the accumulation of intracellular calcium which causes neuron depolarisation [11]. Neurons cultured in a medium without Mg^2+^ for 3 hours generate a neuronal firing frequency of 5∼17 Hz. Furthermore, more than 90% of the neurons continue to undergo spontaneous epileptiform discharges up to 24 hours after replacing the Mg^2+^-free medium with normal medium. This *in vitro* epileptic model has been widely used for investigation of the biochemistry, electrophysiology and molecular biology of the changes that occur under experimental conditions [11], [40], [41], [46]. Therefore, this *in vitro* model was used for this study.

### 4.2. GLP inhibits abnormal calcium accumulation in epileptic hippocampal neurons

The normal extracellular calcium around neuronal cells is in the range of 1.2∼1.3 mmol/L. The calcium concentration in the cell lies in the range of 50∼150 nmol/L. The homeostasis of calcium in the cell and the calcium differential between the external and internal environments of the neuron cell is important for maintaining the basic physiological functions [11]. Intracellular calcium (Ca^2^^+^_i_) homeostasis is maintained by intracellular calcium stores uptake, release and membrane transport processes [11], [45] by receptor-gated calcium channels and voltage-dependent calcium channels, and Ca^2^^+^ is transferred into the cell. At the same time, IP3 can promote the release of Ca^2^^+^ from the cell cytoplasm mitochondria, Golgi apparatus and endoplasmic reticulum. Ca^2^^+^ efflux from neurons is primarily through a calmodulin-dependent Na^+^/Ca^2^^+^ exchanger and ATPase.

Actively opened calcium channels will increase internal Ca^2+^ concentration and increase depolarization of the membrane potential [45]. Hippocampal [Ca^2+^]i levels have been found to gradually increase, up to a year, in the whole animal pilocarpine model of epileptogenesis and also in the neurons of the pilocarpine model of temporal lobe epilepsy [9]. This enhances the presynaptic release of glutamate and further enhances excitability resulting in seizures [11], [45]. The higher concentration of internal calcium stimulates intracellular hydrolase, endonuclease and protein kinase which can induce dysfunction of mitochondria resulting in increased permeability of the mitochondrial membrane, increasing permeability of small ions and molecules and decreasing the membrane potential [11], [45]. This can cause swelling of the mitochondria, which affects the ability to generate ATP, resulting in cell damage affecting its function, which can ultimately lead to cell death.

The high temporal resolution of the laser scanning confocal microscope was applied to monitor the dynamic changes of free Ca^2^^+^_i_ levels using the Ca^2^^+^ fluorescent indicator, Fluo-3/AM [36]. Fluo-3/AM, a fluorescent dye, can penetrate the membrane of the cell. Fluo-3 was used as it is a longer wavelength fluorescent calcium indicator, which can be tested under argon ion laser excitation at 488 nm. In the absence of calcium, Fluo-3 does not generate fluorescence. However, in the presence of Ca^2^^+^, the fluorescence (526 nm) intensity of Fluo-3 was increased by at least 40 times. The fluorescence intensity of Fluo-3/AM does not change with an increase of calcium concentration. After Fluo-3/AM has entered the cell, Fluo-3 is separated from AM by digestion with intracellular esterase and remains inside the cell. Fluo-3, when combined with calcium ions, can produce strong correlative fluorescence. Therefore, measuring the fluorescence intensity reflects the concentration of Ca ^2^^+^_i_ inside the neurons.

This study showed that Ca^2^^+^ fluorescence intensity in the hippocampal neurons in Model group I was significantly higher than that observed in Control group I, soon after the culture medium was replaced with Mg^2+^ free medium, reaching a maximal peak in only 30 s. These results indicate Ca^2^^+^_i_ content increases during the development of epilepsy, but the exact mechanism for the changes still requires further investigation. The peak of intracellular calcium fluorescence intensity in GLP group I was significantly lower compared to the Model group I. Intracellular calcium fluorescence intensity in both GLP group I and Model group I decreased quickly following the peak, but calcium levels in both groups remained higher than the baseline level, with the fluorescence intensity in GLP group I always lower than the fluorescence intensity in Model group I. The results showed that GLP can affect epileptic hippocampus Ca^2^^+^ concentration by inhibiting calcium overload thus preventing an epileptic episode induced by Mg^2+^ deficiency. The mechanism by which GLP affects this change needs to be further investigated. The results also demonstrated that Ca^2^^+^ fluorescence intensity in Model group II, of epileptic hippocampal neurons, was significantly higher than it was in the Control group II, and that there was no significant change after the normal medium was replaced with Mg^2+^ free medium.

Ca^2^^+^ fluorescence intensity in GLP II group, wherein GLP was added into the Mg^2+^ free medium, was significantly lower compared to Model group II. This indicates GLP has a preventative or even therapeutic effect in this system. The mechanism of this effect requires further investigation, but one potential way it may act is through scavenging free radicals, improving and protecting membrane stability [25], [26].

### 4.3 GLP inhibits CaMK II α expression in epileptic hippocampal neurons

CaMK II is involved in the regulation of neuronal activities [17]–[19]. CaMK II α factor plays an important role in Ca^2^^+^ information transfer in many types of neurons. It can bind Ca^2+^ and form a Ca^2+^/CaM complex [20], [21]. Pentylenetetrazol-induced convulsive seizures, even lasting less than a minute, cause translocation of CaMK II α-subunit from the particulate to the soluble fraction for several hours [22]. Intracellular free and non-active CaM binding with an overloading influx of Ca^2+^ will affect the spatial structure and further promote the formation of Ca^2+^/CaM complex, which can then act on targeted enzymes, e.g. phosphodiesterase, triggering a series of physiological or pathological responses [47]. The activated Ca^2+^/CaM complex works with a variety of intracellular CaM binding proteins to induce the excitation and inhibition disorders seen in the central nervous system, causing clinical seizures. This has been explored in a number of experimental models of epilepsy [21], [37], [40]. CaMK II inactivation may modify neuronal cell survival after seizure. In kainic acid-induced status epilepticus in rats CaMK II was decreased 35% in the hippocampus and 20% in the parietal cortex. After 24 h of recovery from kainic acid-induced status epilepsy, all such changes had disappeared [20].

In the hippocampal neuronal culture model of low Mg^2+^ -induced spontaneous recurrent epileptiform discharges, the decrease in CaMK II activity involves a Ca^2+^/N-methyl-D-aspartate (NMDA) receptor-dependent pathway, and a significant decrease in Ca^2+^/calmodulin-dependent substrate phosphorylation of the synthetic peptide autocamtide-2 has been demonstrated. Reduction of extracellular Ca^2+^ levels (0.2 mM in treatment solution) was shown to block the low Mg^2+^-induced decrease in CaMK II-dependent substrate phosphorylation [21]. By reducing the phosphorylation, CaMK II α activity was decreased, such that catecholamine synthesis and secretion decreased and neuronal excitability increased; the GABA_A_ receptor membrane phosphorylation level decreased, so CaMK II α activity decreased, causing neuronal excitability.

During Ca^2^^+^ overload, excessive Ca^2^^+^ influx into the cell occurs activating Ca^2^^+^/CaM. The result of this is a significant increase in CaM expression and inhibition of CaMK II α activity. A reduction in CaMK II α activity can lead to the onset of epilepsy. The results revealed CaMK II α expression in neuronal cells from every group. In the model group, this expression was lower than that observed in the control group, and these findings are consistent with previous studies, suggesting that decreased CaMK II α may play an important role in the generation of seizures, but the specific pathway and detailed mechanism is still to be elucidated. In this study, CaMK II expression in GLP group I was higher than that in the model group, demonstrating some protective or preventative effect, but it was lower than that seen in the normal control group; the fluorescence intensity of CaMK II α in GLP II group was higher than that in the model group, but it was lower than that in the control group.

In summary, these results demonstrate that GLP could inhibit the Ca^2+^ accumulation in neurons and subsequent stimulation of CaMK II α expression, which indicates a beneficial role for GLP in the prevention or treatment of epilepsy.

## Supporting Information

Image S1 The morphology of hippocampal neurons (X200). Neurons were cultured for 24 hours (1A), 3 days (1B), 5 days (1C), and 9 days (1D) respectively. With increased culture time, neurons showed stretched out neurites (day 1), which connected into a network (day 3), and increased somas and dense neurites which were thick and long (day 5). At day 9, the neurons aggregated into clumps, which were unevenly distributed, and it was difficult to identify a single neuron, and were assessed as mature.
(TIF)

Image S2 Calcium distribution in the cytoplasm and nucleus in neurons in Control group I (2A); Model group I (2B) and GLP I (2C) at 30 seconds (X200).
(TIF)

Image S3 Calcium distribution in cytoplasm and nucleus in neurons (X200) in Model group II at 0 (3A) and 180 (3B) minutes; in GLP group II at 0 (3C) and 180 (3D) minutes.
(TIF)

Image S4 CaMK II α protein was expressed in Control group III (4A), Model III (4B), GLP III (4C) and IV (4D) hippocampal neurons.
(TIF)
