# Correction: Anti-Epileptic Effect of Ganoderma Lucidum Polysaccharides by Inhibition of Intracellular Calcium Accumulation and Stimulation of Expression of CaMKII α in Epileptic Hippocampal Neurons

**DOI:** 10.1371/journal.pone.0111295

**Published:** 2014-10-10

**Authors:** The PLOS ONE Staff

“Normal group” should read “Control group” in Figures 1, 2, 3 and 4. Figure 4 is incorrect, only Figure 4A was published and Figure 4B was incorrectly omitted. The authors have provided a corrected version of Figures 1, 2, 3 and 4 here.

**Figure 1:**
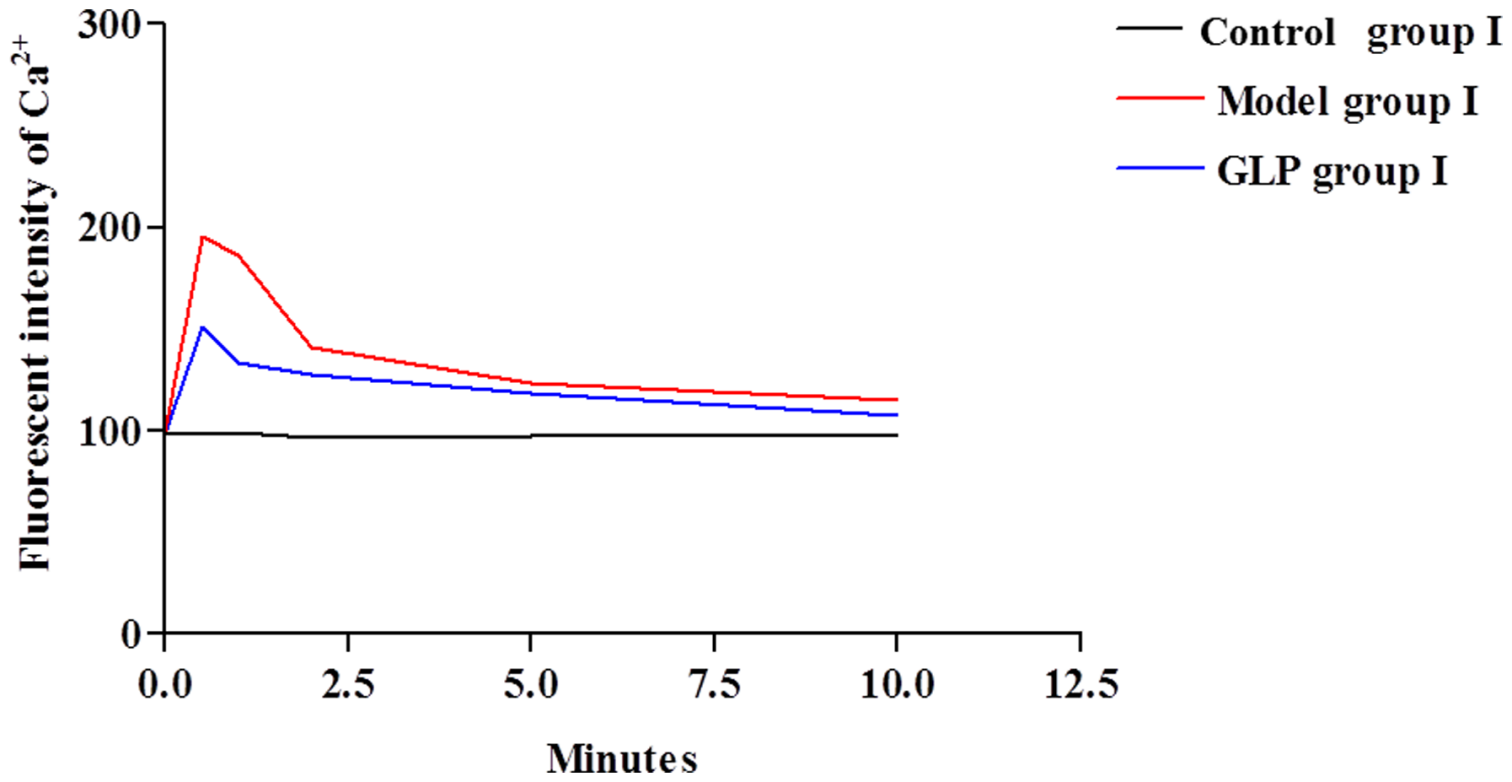
Changes in fluorescence intensity of Ca^2+^ in neurons during establishment of the model, n  =  4, thirty neurons were observed in each replicate. There is a significant increase (within 30 seconds) in fluorescence intensity of Ca^2+^ in neurons in the model group compared to the control group (p<0.001). This increase also occurs in the GLP I group, and it decreases in both Model and GLP I groups with time up to 10 minutes when the Model group is still significantly higher than the Control group I, but, there is no difference between the Control group I and GLP I groups at 10 minutes. Tukey’s test following one way ANOVA was used to perform statistical analysis.

**Figure 2:**
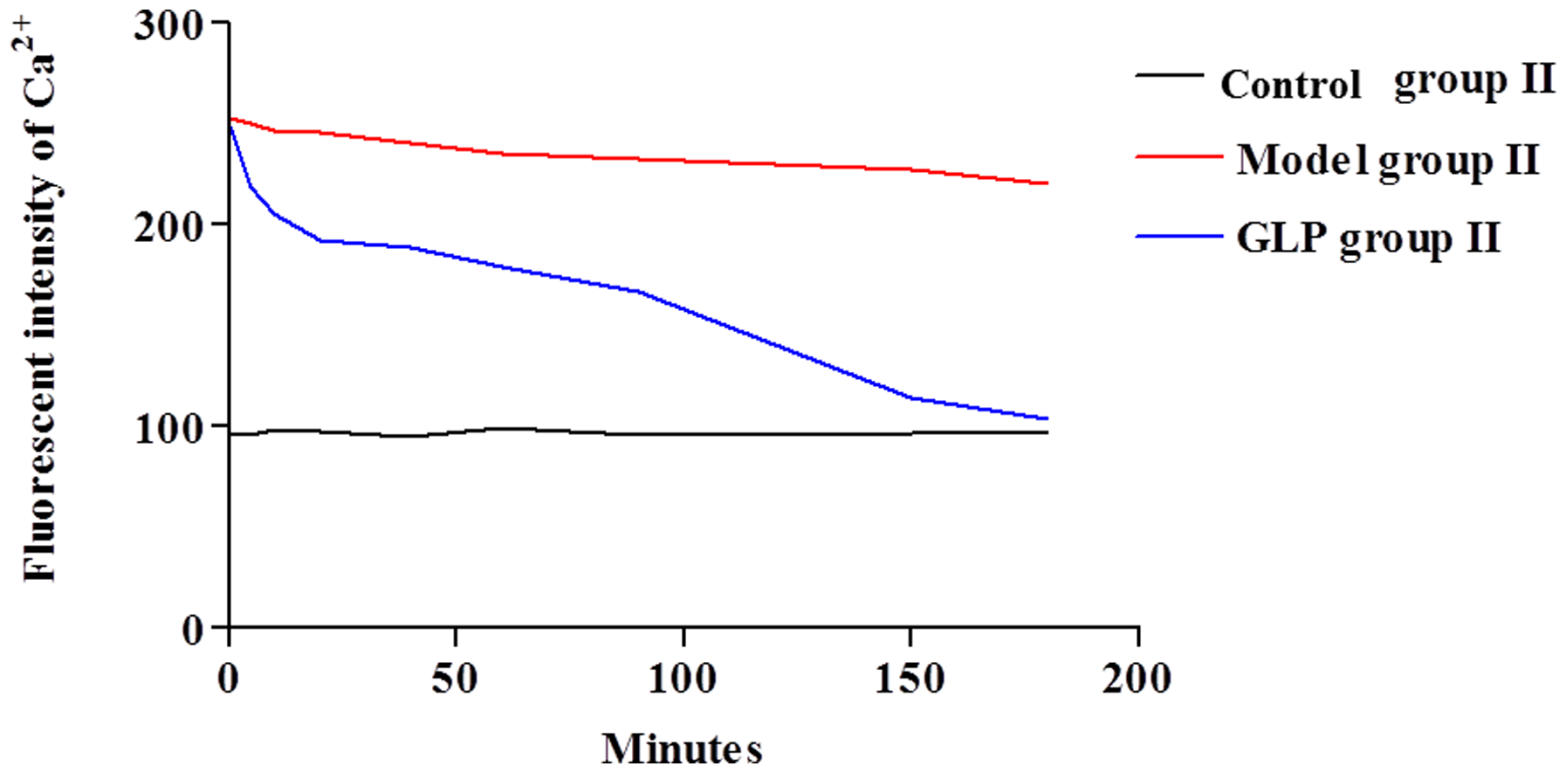
Changes in fluorescence intensity of Ca^2+^ in neurons during the treatment, n  =  4, thirty neurons were observed in each replicates. There is no change in fluorescence intensity of Ca^2+^ in neurons in the Control group II during a 3 hour period. Fluorescence intensity of Ca^2+^ in neurons was highest in neurons cultured in Mg^2+^ ion free medium for 3 hours prior to replacement with normal maintaining medium i.e. Model group II; there is no significant decrease (P>0.05) between the zero time point (0 hour) and 3 hours following the normal medium replacement. However, following GLP treatment, there is a gradual decrease in Fluorescence intensity of Ca^2+^ in neurons, with levels almost returning to normal after 3 hours. Tukey’s test following one way ANOVA was used to perform statistical analysis.

**Figure 3:**
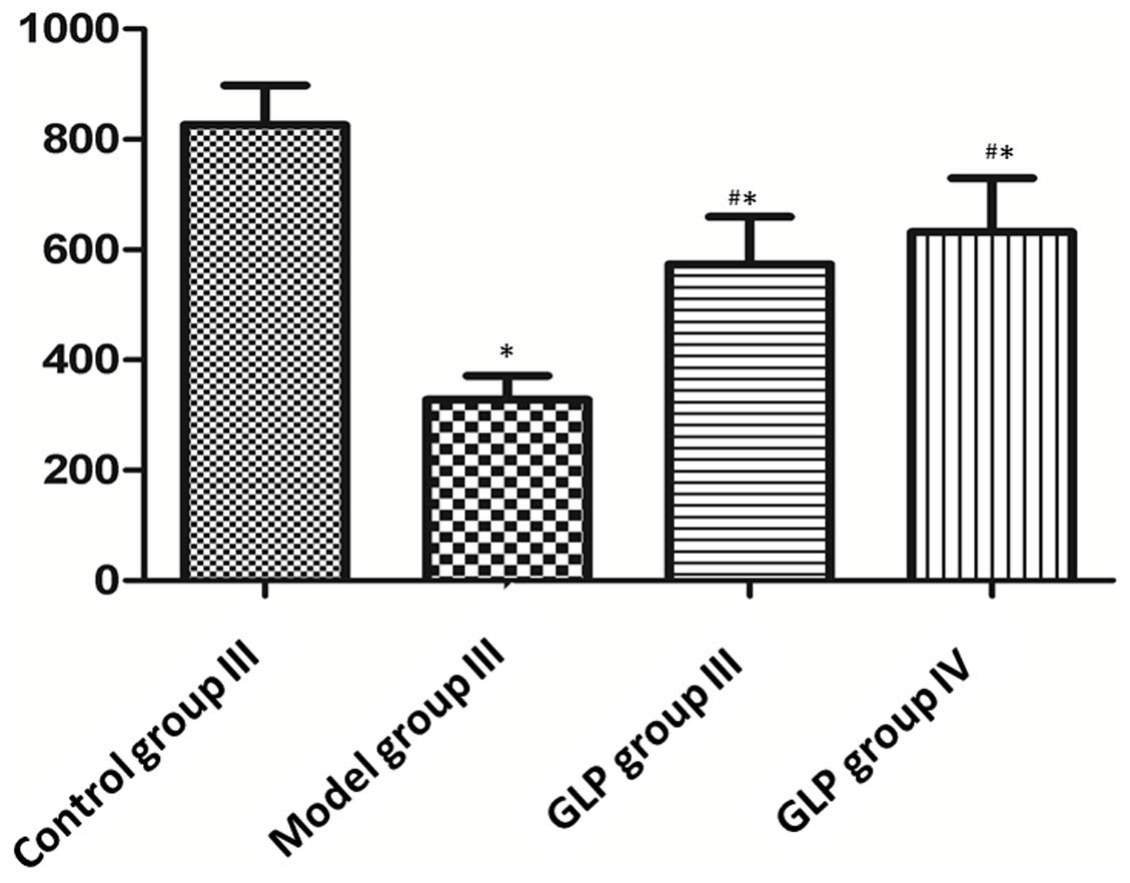
The fluorescence intensity of CaMK II α protein in hippocampal neurons in Control group III (A), Model group III (B), GLP group III (C) and IV (D). Data shown as mean±SD, n  =  4. Corresponding, representative images are shown in Images S4A, S4B, S4C and S4D. Tukey’s test following one way ANOVA was used to perform the statistical analysis. #compared to Control group III, p<0.01; *compared to Model group III, p<0.01.

**Figure 4:**
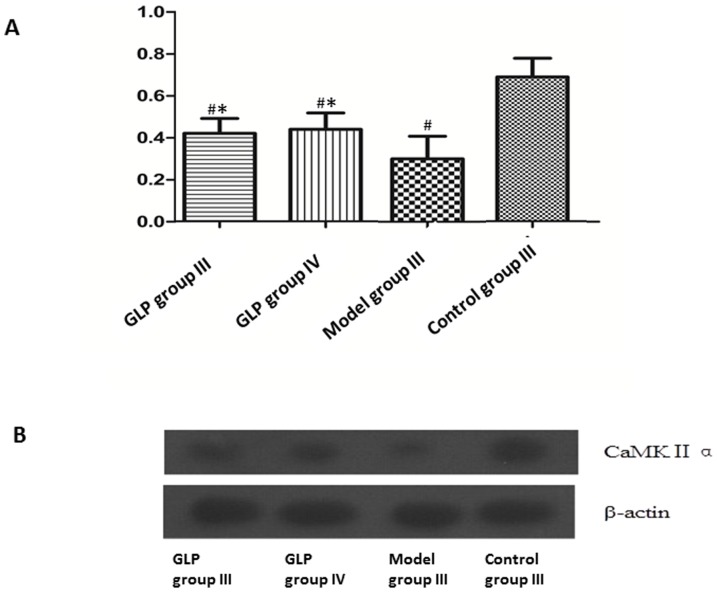
CaMK II α protein expression. Relative amount of CaMK II αprotein expressed in neurons in different groups (A) and Western blot analysis (B) of neurons from each untreated/treated group. Data in figure 4A is shown as mean±SD, n  =  4. Tukey’s test following one way ANOVA was used to perform the statistical analysis. #indicated p<0.01, compared to Control group III; *indicated p<0.01, compared to Model group III.
