# Supplementary figures and images for: Anti-Epileptic Effect of Ganoderma Lucidum Polysaccharides by Inhibition of Intracellular Calcium Accumulation and Stimulation of Expression of CaMKII α in Epileptic Hippocampal Neurons
Source: PLoS One. 2014 Jul 10;9(7):e102161. doi: 10.1371/journal.pone.0102161 (PMC4092074; doi:10.1371/journal.pone.0102161)

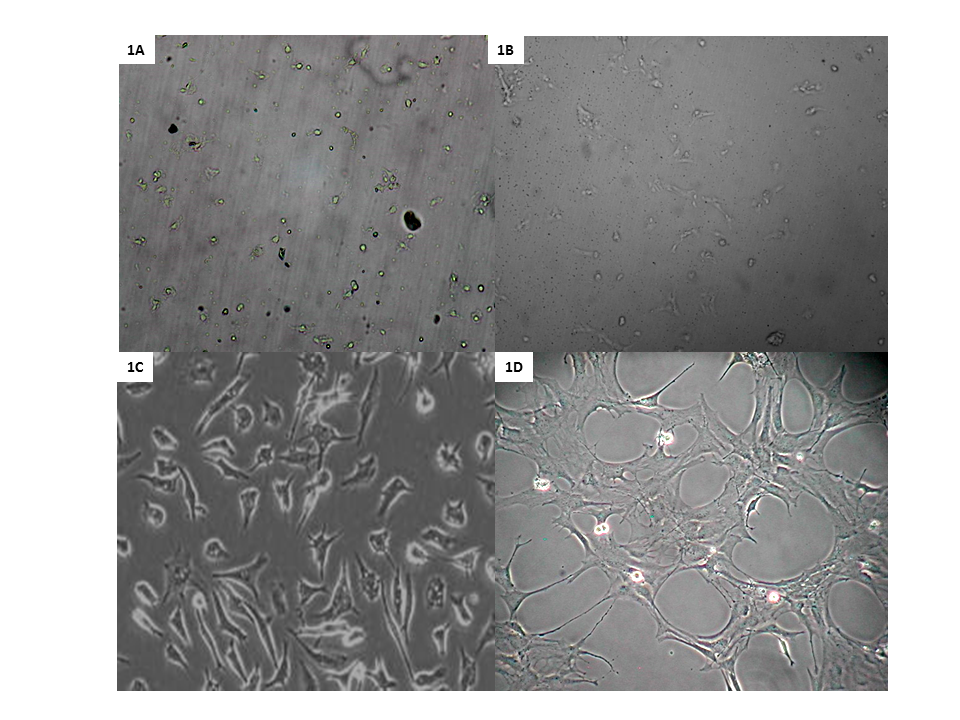

Supplement: Image S1 — The morphology of hippocampal neurons (X200). Neurons were cultured for 24 hours (1A), 3 days (1B), 5 days (1C), and 9 days (1D) respectively. With increased culture time, neurons showed stretched out neurites (day 1), which connected into a network (day 3), and increased somas and dense neurites which were thick and long (day 5). At day 9, the neurons aggregated into clumps, which were unevenly distributed, and it was difficult to identify a single neuron, and were assessed as mature. (TIF) [file pone.0102161.s001.tif]

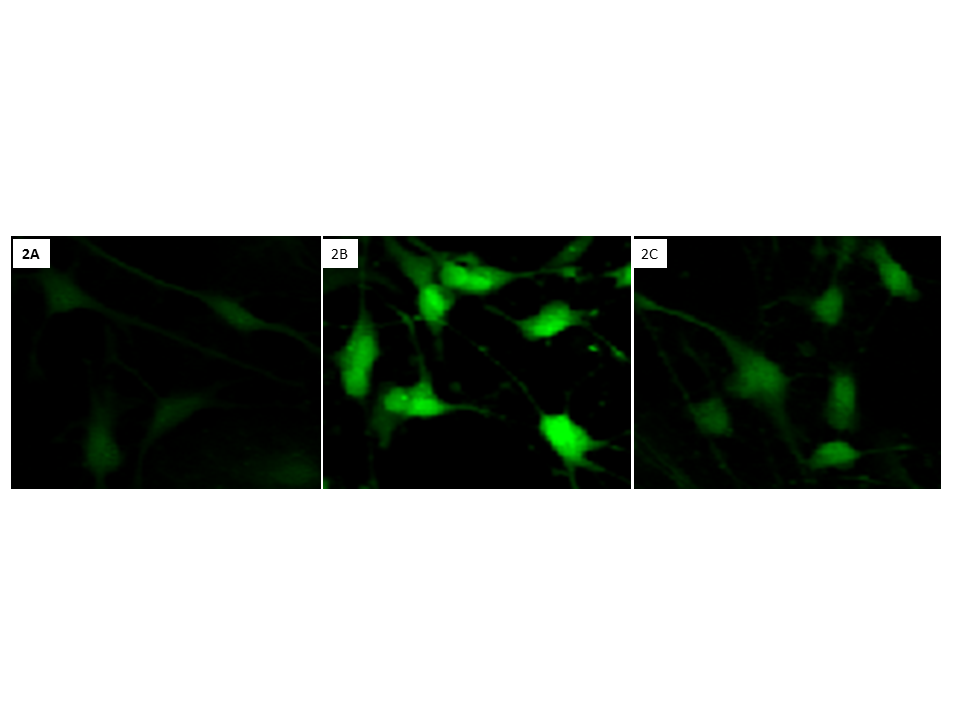

Supplement: Image S2 — Calcium distribution in the cytoplasm and nucleus in neurons in Control group I (2A); Model group I (2B) and GLP I (2C) at 30 seconds (X200). (TIF) [file pone.0102161.s002.tif]

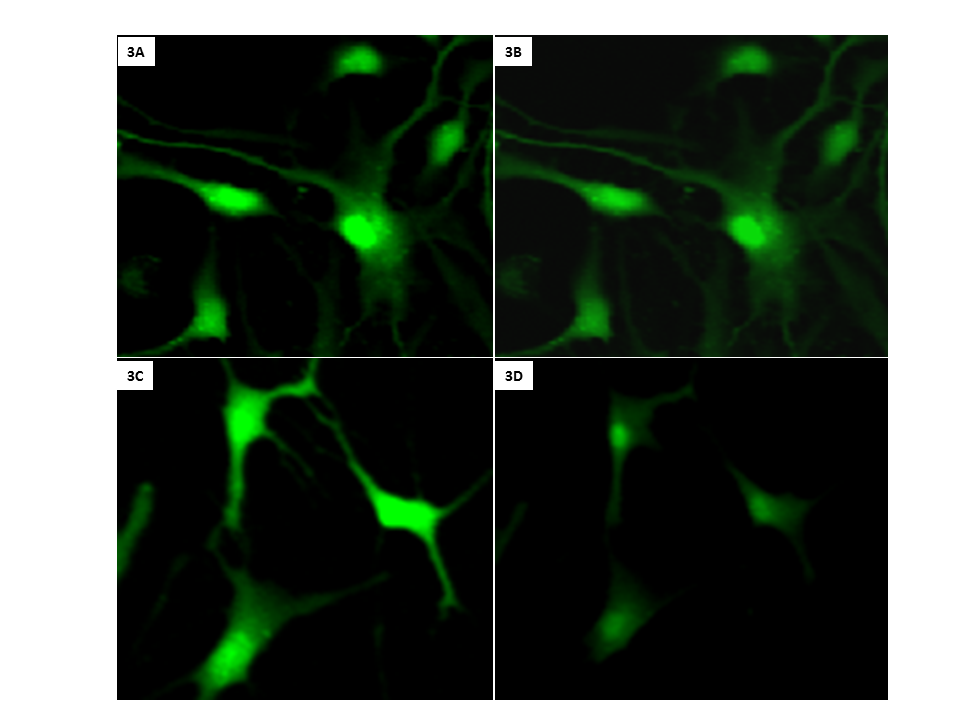

Supplement: Image S3 — Calcium distribution in cytoplasm and nucleus in neurons (X200) in Model group II at 0 (3A) and 180 (3B) minutes; in GLP group II at 0 (3C) and 180 (3D) minutes. (TIF) [file pone.0102161.s003.tif]

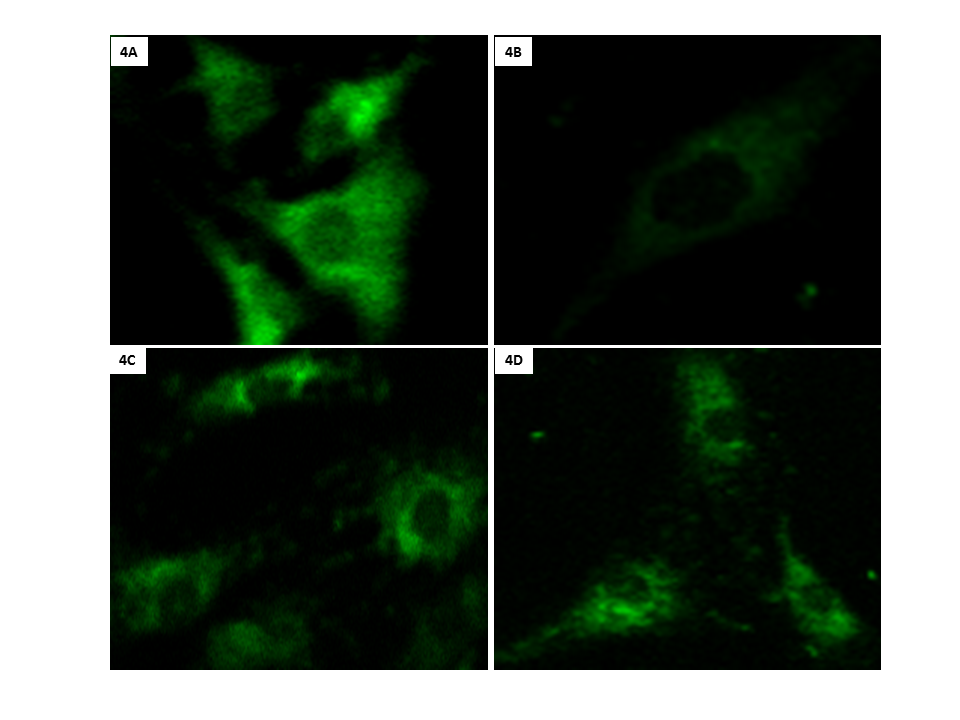

Supplement: Image S4 — CaMK II α protein was expressed in Control group III (4A), Model III (4B), GLP III (4C) and IV (4D) hippocampal neurons. (TIF) [file pone.0102161.s004.tif]
